# A Cost-Effective Hydrogel-Based Wound Model for Quantitative Evaluation of Topical Treatments in Infected Wounds

**DOI:** 10.3390/gels12100934

**Published:** 2026-10-16

**Authors:** Amalie Kjær Andresen, Emilia Gaertner, Marie Karen Tracy Hong Lin, Claus Moser, Lars Jelsbak, Line Hagner Nielsen

**Affiliations:** 1 Department of Health Technology, Technical University of Denmark, Ørsteds Plads 345C, 2800 Kongens Lyngby, Denmark; 2 National Centre for Nano Fabrication and Characterization, Technical University of Denmark, Fysikvej 307, 2800 Kongens Lyngby, Denmark; mktracy@dtu.dk; 3 Department of Clinical Microbiology, Rigshospitalet, Copenhagen University Hospital, Blegdamsvej 9, 2100 Copenhagen East, Denmark; moser@dadlnet.dk; 4 Department of Immunology and Microbiology, Costerton Biofilm Center, University of Copenhagen, Blegdamsvej 3B, 2100 Copenhagen East, Denmark; 5 Department of Biotechnology and Biomedicine, Section for Microbial and Chemical Ecology, Technical University of Denmark, Søltofts Plads 221, 2800 Kongens Lyngby, Denmark; lj@bio.dtu.dk

**Keywords:** *in vitro* wound model, biofilm, antimicrobial evaluation, alginate–gelatin hydrogel, *Pseudomonas aeruginosa*, transwell system, simulated wound fluid, antibiofilm

## Abstract

Chronic wound infections are frequently associated with bacterial biofilms, which significantly reduce the efficacy of antimicrobial treatments. Conventional *in vitro* evaluation methods, such as disc diffusion assays and static biofilm models, lack physiological relevance, whereas more advanced wound models are often costly, complex, and difficult to reproduce. In this study, we present a cost-effective and easily applicable *in vitro* wound model based on gelatin–alginate hydrogels for the evaluation of topical antimicrobial therapies. Hydrogels composed of gelatin type A and sodium alginate were integrated into a transwell system and crosslinked using microbial transglutaminase and calcium ions. This configuration enables passive diffusion of simulated wound fluid from the basolateral compartment, thereby mimicking key aspects of the wound environment. The model supports the growth of *Pseudomonas aeruginosa* biofilms within a three-dimensional matrix. The hydrogel system supported bacterial growth and microcolony formation during 48 h of incubation at 37 °C. The system was applied in both biofilm inhibition and eradication studies using tobramycin, ε-poly-L-Lysine and the commercially available wound gel Prontosan^®^. Viable bacteria were quantitatively recovered through hydrogel dissolution followed by serial dilution and count of colony-forming units. Overall, the developed model provides a simple and accessible platform for quantitative evaluation of antibiofilm treatments. While not intended to fully replicate the complexity of native tissue, this system offers a practical alternative to collagen-based models and helps bridge the gap between conventional *in vitro* assays and more advanced wound models.

## 1. Introduction

Chronic wounds represent a major healthcare burden and are frequently complicated by bacterial biofilms, which are increasingly recognized as one of the primary factors preventing wound healing [1,2,3,4]. In contrast to planktonic bacteria, biofilm-associated microorganisms exhibit increased tolerance to antimicrobial treatment and host immune responses, making infections difficult to eradicate [5]. Consequently, topical antimicrobial therapies, including wound dressings, gels, and antimicrobial polymers, have become increasingly important for managing localized wound infections while reducing the need for systemic antibiotics [6].

Despite the growing development of topical antimicrobial formulations, there is currently no universally accepted *in vitro* model for evaluating their efficacy under physiologically relevant wound conditions. Instead, antimicrobial wound dressings are commonly assessed using agar diffusion assays, the minimum inhibitory concentration (MIC) testing of extracted compounds, or biofilms grown in conventional well plates [7]. Although these methods are simple and highly reproducible, they fail to mimic the biological and physical characteristics of chronic wound biofilms. Thus, they provide limited information regarding the performance of topical treatments *in vivo*.

The architecture of chronic wound biofilms differs substantially from the conditions represented by conventional laboratory assays. Rather than growing on rigid surfaces such as glass, plastic, or agar, wound biofilms develop within a semi-solid tissue matrix located at the interface between air and wound tissue, where nutrients are supplied from the underlying wound bed through wound exudate [1]. Furthermore, chronic wounds are colonized by multiple microbial species and exhibit heterogeneous gradients of oxygen, nutrients, and metabolic activity throughout the biofilm [8]. These characteristics influence both biofilm development and antimicrobial susceptibility and should therefore be considered when designing *in vitro* wound models.

Several advanced wound biofilm models have been developed to better mimic the wound microenvironment. Examples include the flat-bed perfusion model [9], the collagen-based wound model [10], the agarose capillary tube model [11], the Duckworth Biofilm Device (DBD) [1], and more recently layered collagen wound models [12]. While these systems have substantially improved physiological relevance, they require either specialized equipment, continuous perfusion systems, complex multilayer fabrication, or expensive collagen matrices. Consequently, many of these models are difficult to reproduce and implement in routine laboratory settings, limiting their widespread usage. As a result, researchers frequently continue to rely on simpler screening methods despite their limited physiological relevance.

In this study, we present a simple, cost-effective hydrogel-based wound model for quantitative evaluation of topical antimicrobial treatments against biofilm-associated wound infections with a focus on *Pseudomonas aeruginosa*. When designing our model, emphasis was placed on reproducing key functional characteristics of chronic wounds while maintaining simplicity and affordability. Based on current recommendations for wound biofilm models [8], the system was designed to (I) utilize simulated wound fluid (SWF) as the nutrient source, (II) provide passive nutrient transport through a transwell configuration rather than continuous shear flow, (III) support three-dimensional biofilm growth within a hydrated host matrix, and (IV) maintain a largely static environment resembling the wound bed.

To improve accessibility compared with collagen-based systems, the host matrix consists of gelatin and sodium alginate crosslinked using microbial transglutaminase (mTG) and calcium ions. Gelatin was selected as a matrix component because of its relatively low cost, biological origin, and ease of processing. Gelatin is obtained through the hydrolysis of collagen, with acid-mediated processing resulting in gelatin type A (GA) used in the present study. Its collagen-derived nature provides a biologically relevant matrix component, while its accessibility and low cost make it attractive for development of a simple and cheap wound model [13]. Furthermore, mTG enables the enzymatic crosslinking of GA and was selected as a comparative mild alternative to conventional chemical crosslinking strategies [13].

Alginate was selected as a complementary polymer because of its likewise low cost, availability, and ability to undergo rapid ionic crosslinking with divalent cations such as Ca^2+^, thereby contributing to hydrogel structural stability. However, alginate lacks the cell-adhesion motifs associated with naturally derived extracellular matrix components and therefore has limited biological functionality when used as a sole matrix material [14]. Combining alginate with gelatin can therefore provide complementary properties, with alginate contributing structural stability and gelatin providing a collagen-derived component with greater biological similarity to native tissue.

The gelatin–alginate combination was therefore selected to provide a balance between structural stability and biological characteristics while maintaining a simple and cost-effective formulation. The use of mTG and Ca^2+^ as a complementary crosslinking mechanism was intended to stabilize the gelatin and alginate components, respectively. This combination was considered suitable for the present model, where the hydrogel must maintain sufficient integrity during incubation while providing a hydrated three-dimensional environment that supports bacterial growth and subsequent quantitative recovery.

**Figure 1:**
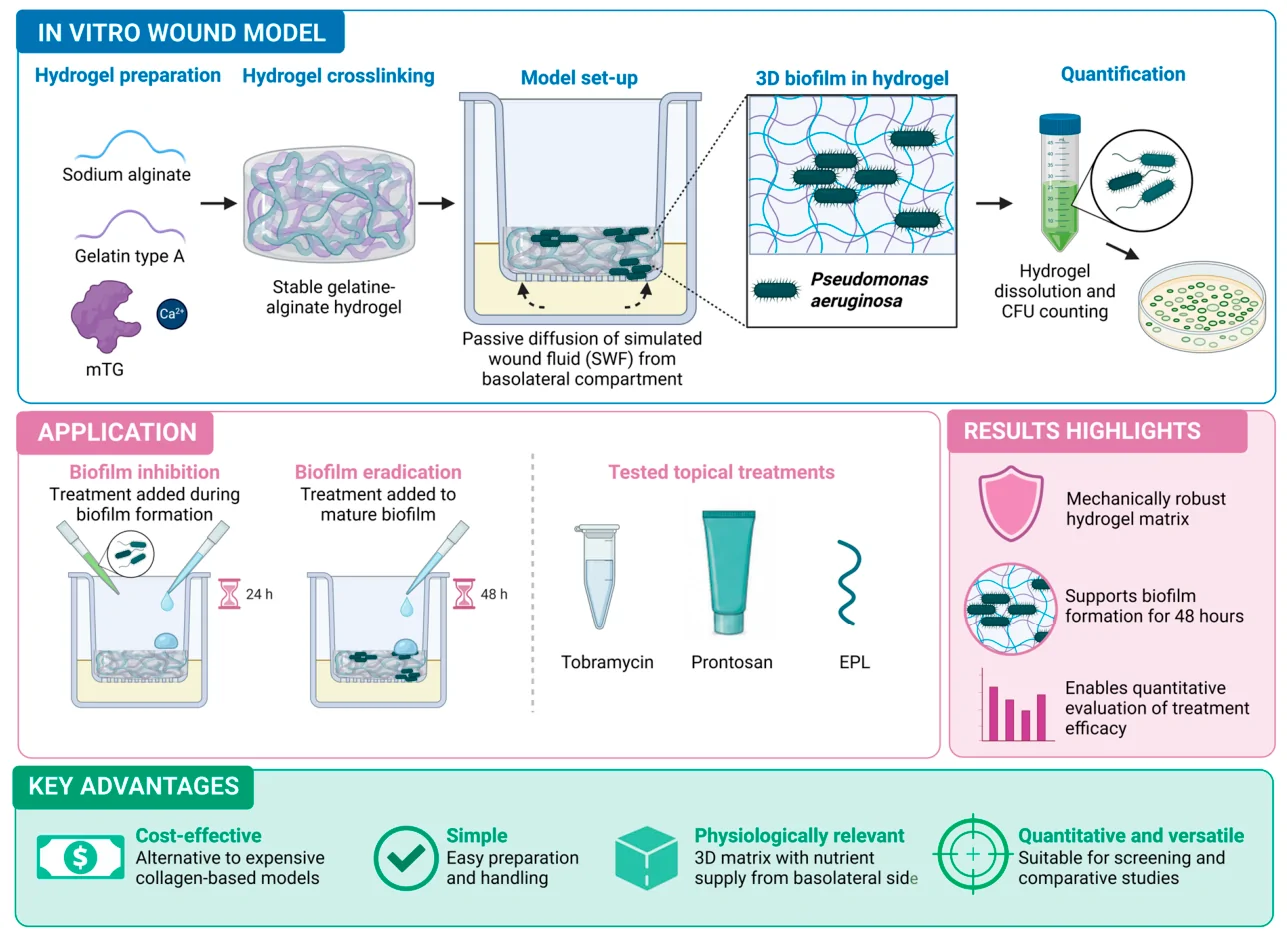
Schematic illustration of the fabrication of the hydrogel wound model and its application for evaluating antimicrobial efficacy against *P. aeruginosa* biofilm growth inhibition and eradication. The illustration depicts the experimental set-up and the resulting hydrogel model.

The architecture of the established model is illustrated in Figure 1. The hydrogel is formed directly within 24-well transwell inserts through an initial thermal crosslinking step followed by enzymatic and ionic crosslinking. The inserts are subsequently placed in 24-well plates containing SWF in the basolateral compartment. This configuration separates the hydrogel from the nutrient-containing fluid while allowing for the passive transport of nutrients through the porous membrane from below.

The model was validated using the antibacterial polymer ε-poly-L-lysine (EPL), the marketed antibiotic tobramycin (TOB), and the commercially available wound gel Prontosan^®^, demonstrating its applicability for both biofilm inhibition and eradication studies [15,16].

## 2. Results and Discussion

### 2.1. Hydrogel Stability Evaluation

Hydrogels based on natural biopolymers, such as the polysaccharide alginate and proteins such as gelatin, provide biocompatible matrices that can support microbial colonization and biofilm development [17]. However, their susceptibility to hydrolytic and enzymatic degradation can limit their long-term structural stability [18]. Gelatin is obtained through the hydrolytic extraction of collagen, resulting in either type A gelatin, produced under acidic conditions, or type B gelatin, produced under alkaline conditions. In the present study, type A gelatin was selected due to its amino acid composition and suitability for enzymatic crosslinking by mTG, which catalyzes covalent crosslinking between glutamine and lysine residues. This additional crosslinking was expected to improve the structural stability of the gelatin matrix and reduce its susceptibility to enzymatic degradation [19,20,21].

Sodium alginate was incorporated to further enhance the structural stability of the hydrogel. Alginate has been extensively used as a matrix for *P. aeruginosa* inoculation and cultivation for biofilm models [22]. Notably, alginate is also produced by bacteria of the genera *Pseudomonas* and *Azotobacter* and constitutes an important component of the extracellular polymeric substance (EPS) in biofilms [23]. Upon ionic crosslinking with divalent cations such as Ca^2+^, alginate forms a mechanically stable hydrogel network, making it suitable as a supporting matrix for cell culture and three-dimensional biological models [24].

**Figure 2:**
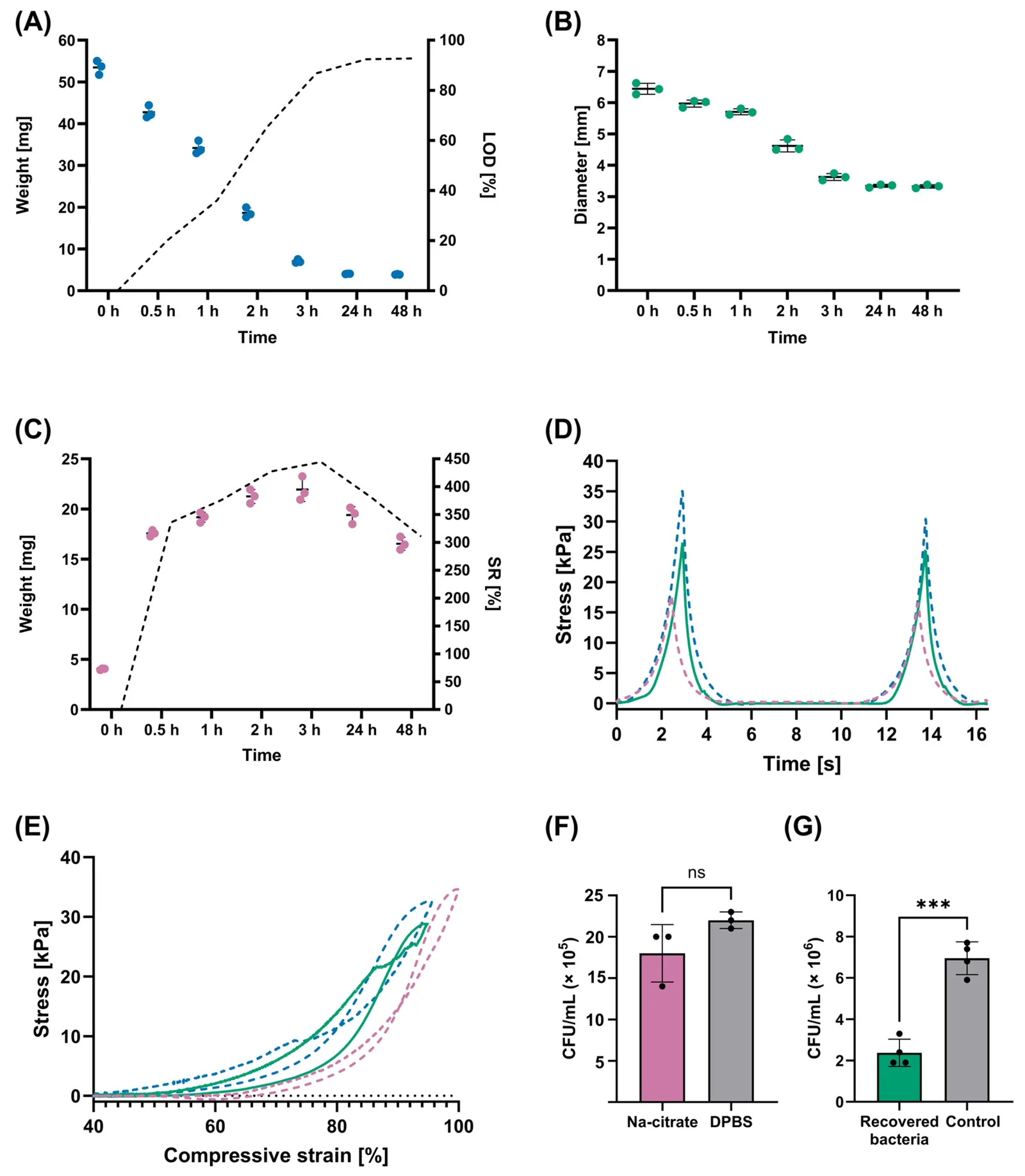
Characterization of hydrogel stability and swelling behavior in the absence of *P. aeruginosa*. (**A**) Loss on drying (LOD) of fully hydrated hydrogels incubated at 37 °C for 48 h. Hydrogel weight is shown in blue, while the dotted line represents LOD (%). (**B**) Change in hydrogel diameter over 48 h at 37 °C for the same samples used for LOD measurements. (**C**) Swelling behavior of hydrogels initially dried at 37 °C for 24 h and subsequently immersed in PBS. Hydrogel weight is shown in pink, while the dotted line represents the swelling ratio (SR; %). (**D**) Uniaxial compression testing of three individual hydrogel samples indicated from three different colors (blue, green, and purple) subjected to two consecutive compression cycles of 50% compressive strain shown as time in seconds on x-axis and stress in kPa on y-axis. (**E**) Compression of three individual hydrogel samples indicated from three different colors (blue, green, and purple) until mechanical failure, shown as stress–strain curves. (**F**) Evaluation of trisodium citrate (Na-citrate) toxicity toward *P. aeruginosa*. Bacterial viability following incubation in Na-citrate was compared with bacteria incubated under the same conditions in DPBS and quantified by colony-forming unit (CFU) enumeration. (**G**) Bacterial recovery efficiency of the hydrogel-based wound model relative to the direct-plating control. Data are presented as means ± SDs; *** = *p* < 0.0001; *n* = 3–4.

The stability and hydration behavior of the hydrogel at 37 °C were first investigated prior to bacterial inoculation by evaluating loss on drying (LOD), changes in diameter, and swelling ratio (SR). These measurements were performed as an initial physicochemical characterization to assess the ability of the hydrogel to retain its structure and evaluate its hydration capacity. The LOD analysis (Figure 2A) showed a pronounced reduction in hydrogel weight from 53.5 ± 1.7 mg at 0 h to 3.9 ± 0.1 mg at 48 h, corresponding to an LOD of 92.7%. Notably, 86.8% of the initial mass was lost within the first 4 h, indicating that the majority of water loss occurred during the initial drying period. Consistent with this observation, the hydrogel diameter (Figure 2B) decreased from 6.4 ± 0.2 mm at 0 h to 3.3 ± 0.1 mm after 48 h, with the majority of the dimensional change occurring within the first 3 h. The subsequent stabilization in diameter suggests that the hydrogel reached a relatively stable dehydrated state at 37 °C. Importantly, no increase in diameter or apparent macroscopic structural collapse associated with thermal melting was observed during incubation, indicating that the crosslinked hydrogel maintained overall structure under the conditions used for subsequent biofilm experiments. The relatively high gelatin-to-alginate ratio in the formulation (5% and 2% (*w*/*v*), respectively) may contribute to the observed behavior, as gelatin constitutes the major polymeric component of the hydrogel.

The swelling behavior of the hydrogel was subsequently evaluated (Figure 2C). The hydrogel exhibited a high initial swelling ratio, reaching 444.7 ± 39.6% after 3 h of incubation in PBS. The swelling ratio subsequently decreased to 381.5 ± 28.1% after 24 h and 310.7 ± 23.7% after 48 h. Thus, although the hydrogel demonstrated high water uptake capacity, a gradual reduction in swelling was observed during prolonged incubation. This behavior suggests that the hydrogel does not retain all the initially absorbed water over extended periods, potentially reflecting polymer rearrangement, a possible decrease in crosslinking degree, or partial loss of soluble components [25]. Previous studies have similarly reported that increasing the gelatine-to-alginate ratio can enhance the swelling capacity of gelatin–alginate hydrogel systems [26,27,28]. Furthermore, the relatively stable swelling observed between 30 min and 24 h is consistent with previously reported gelatin–alginate hydrogel systems [26]. Nevertheless, the substantial swelling capacity observed throughout the 48 h incubation period indicates that the hydrogel retains a considerable aqueous phase, which may support diffusion of nutrients and metabolites within the matrix. This can be beneficial for three-dimensional cell and microbial models by facilitating transport of nutrients and metabolites [29]. Therefore, the observed swelling behavior was considered suitable for supporting *P. aeruginosa* biofilm growth within the hydrogel matrix.

Uniaxial compression testing was performed to characterize the mechanical response of the hydrogel matrix (Figure 2D). The hydrogels were subjected to two consecutive compression cycles at 50% strain. All three samples exhibited comparable peak stress between the first and second compression cycle. Peak stress values during the first compression cycle were 35.0, 25.9, and 17.9 kPa for the three individual samples, compared with 30.3, 24.9, and 16.8 kPa during the second cycle, respectively. Thus, the second compression cycle corresponds to 86%, 96%, and 94% of the peak stress measured during the first cycle. The relatively similar stress responses between the two cycles indicate that the hydrogels retained most of their compressive resistance following the initial deformation.

The compressive modulus was calculated from the linear regression of each stress–strain curve and was 32, 7.5, and 11 kPa for the three samples, respectively (Figure S1). The variation between samples indicates some sample-to-sample variability in the mechanical response of the hydrogel matrix. Nevertheless, all three samples exhibited a distinct approximate linear region at lower strains, extending to approximately 23–27% compressive strain. The observed mechanical response indicates that the hydrogel possesses measurable resistance to compressive deformation while retaining a similar response following repeated loading.

The measured compressive moduli were within the range reported for several soft gelatin–alginate and other hydrogel systems developed for three-dimensional cell culture [30,31]. For comparison, Ramanova et al. reported a compressive modulus of approximately 2.1 kPa for an alginate hydrogel prepared using 4% (*w*/*v*) alginate and 0.2 M CaCl_2_ [32]. The higher modulus observed in the present study may reflect differences in hydrogel composition, crosslinking conditions, sample dimensions, and testing methodology. Direct comparison between hydrogel systems should be made cautiously, as compressive properties are strongly influenced by these factors. However, the compressive modulus measured in the present study is consistent with a relatively soft hydrogel matrix, which still provides sufficient mechanical resistance to withstand the applied deformation during handling and model preparation.

Compression testing above 50% compressive strain was additionally performed to investigate the mechanical response approaching failure (Figure 2E and Figure S2). Greater variability was observed between samples under these conditions. Two hydrogels exhibited distinct failure points at approximately 9 and 21.6 kPa, whereas the third sample did not display a distinct failure point within the tested compression range. The variation suggested that failure behavior was less consistent between individual hydrogel samples than the response observed during repeated compression to 50% strain.

### 2.2. Model Validation and Optimization

For quantitative recovery of bacteria from the hydrogel matrix, complete dissolution of the hydrogel was required prior to CFU enumeration. During preliminary experiments *P. aeruginosa* appeared to degrade the gelatin component of the matrix during incubation, while the structural integrity of the remainder was attributed primarily to the crosslinked alginate network. Proteolytic degradation of gelatin by *P. aeruginosa* has previously been associated with extracellular protease activity and may contribute to matrix infiltration and bacterial migration [33]. To facilitate dissolution of the remaining alginate network, trisodium citrate dihydrate was employed as a chelating agent due to its ability to complex Ca^2+^ ions and thereby disrupt the ionic crosslinks of the remaining alginate network [34]. Because of the potential effect of trisodium citrate dihydrate on bacterial viability under the conditions used for hydrogel dissolution had not been established, a toxicity control was performed. As shown in Figure 2F, no significant difference in CFU counts was observed between bacteria incubated in trisodium citrate dihydrate and those incubated in DPBS. These results indicate that the citrate treatment did not measurably compromise bacterial viability under the conditions applied and was, therefore, considered suitable for subsequent bacterial recovery from the hydrogel matrix.

The recovery efficiency of the hydrogel dissolution and bacterial recovery procedure was subsequently evaluated (Figure 2G). The mean bacterial concentration recovered from the hydrogel-containing transwell inserts was 2.4 ± 0.7 × 10^6^ CFU/mL, compared with 6.9 ± 0.8 × 10^6^ CFU/mL obtained from the corresponding direct-plating control. This corresponded to a recovery efficiency of 34.2% relative to the control.

The nominal bacterial concentration of the inoculum was approximately 2 × 10^7^ CFU/mL; however, the recovered concentration in both the hydrogel and control groups was lower than the nominal inoculum concentration. Thus, the difference between the nominal inoculum and the enumerated control cannot be attributed solely to the hydrogel recovery procedure and may reflect losses associated with dilution, handling, and sample processing. Therefore, recovery efficiency represents the proportion of bacteria recovered from the hydrogel relative to the corresponding control under the applied experimental procedure.

Importantly, the same recovery procedure was applied to all samples used for the subsequent inhibition and eradication experiments. Although recovery efficiency was below 100%, the consistent application of the procedure provides a standardized basis for comparison of viable bacterial counts between experimental groups. The recovery efficiency should thus be considered when interpreting the absolute CFU values reported in the subsequent experiments.

The concentration of CaCl_2_ used for alginate crosslinking was subsequently optimized to identify suitable overall model conditions. CaCl_2_ concentrations of 1.0, 1.5, 2.0, and 2.5% (*w*/*v*) were evaluated based on the resulting CFU counts of *P. aerugonosa* after 24 h of biofilm growth (Figure S3). The highest CFU/mL was obtained using 1.5% (*w*/*v*) CaCl_2_. Interestingly, this condition resulted in a higher CFU count compared with the 1.0% condition, which may indicate that the increased crosslinking density provided greater structural stability of the hydrogel while still allowing for bacterial growth. In contrast, higher CaCl_2_ concentrations did not result in a further increase in CFU/mL. Excessive crosslinking may produce a denser alginate network that limits bacterial migration or nutrient transport, while residual Ca^2+^ may also influence bacterial viability despite the washing steps included in the protocol [35]. However, in all cases, the recovered CFU counts exceeded the initial inoculation concentration (~10^5^ CFU/mL), suggesting that the selected conditions supported bacterial growth rather than loss of bacterial viability. Based on the overall performance of the hydrogel system, evaluated through CFU enumeration, 1.5% (*w*/*v*) CaCl_2_ was selected as the optimal crosslinking concentration for overall model conditions and was used for all subsequent experiments.

### 2.3. Bacterial Growth Evaluation on Gelatin–Alginate Hydrogels Using SEM

**Figure 3:**
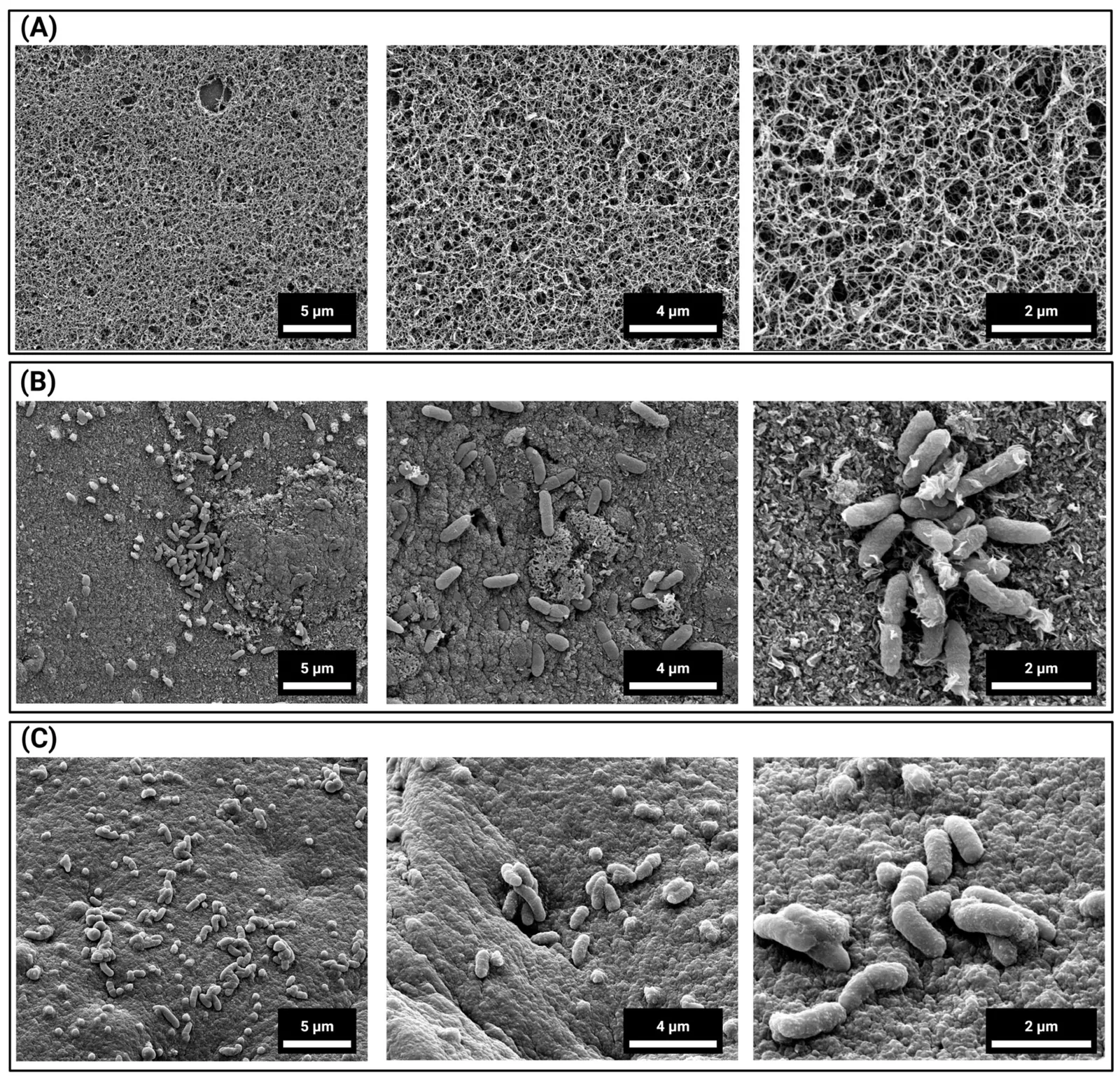
Scanning electron microscopy analysis of bacterial growth and biofilm formation on the apical surface of the hydrogel matrix following critical point drying. Hydrogels were analyzed at (**A**) 0 h without bacterial inoculation, and (**B**) 24 h and (**C**) 48 h following incubation with *P. aeruginosa* wildtype. Images were acquired at different magnifications and positions within the hydrogel matrix. Scale bars are indicated in the respective images.

Prior to evaluating antimicrobial treatment strategies against *P. aeruginosa*, the ability of the hydrogel matrix to support bacterial growth and biofilm development was investigated by scanning electron microscopy (SEM) (Figure 3). Hydrogels without bacterial inoculation were examined to characterize the initial matrix morphology (Figure 3A), while hydrogels incubated with *P. aeruginosa* for 24 and 48 h were analyzed to assess changes in the matrix architecture during biofilm development (Figure 3B,C). The 24 h and 48 h time points were selected to represent the hydrogel conditions used for subsequent biofilm growth inhibition and eradication experiments, respectively.

The non-inoculated hydrogel matrix exhibited distinct porous morphology with a relatively homogeneous network of small micropores (Figure 3A). Such porous architecture is characteristic of gelatin–alginate hydrogel systems and provides an interconnected matrix that can facilitate bacterial infiltration and transport of nutrients and metabolites [24]. The observed morphology is consistent with previous reports of gelatin–alginate hydrogels initially crosslinked with mTG, where the enzymatic crosslinking of the gelatin component results in a homogeneous matrix containing small micropores. The subsequent ionic crosslinking of alginate with Ca^2+^ can further stabilize the polymer network and contribute to the overall structural integrity of the hydrogel [36].

Following 24 h of incubation with *P. aeruginosa*, a pronounced change in the hydrogel morphology was observed (Figure 3B). Compared with the non-inoculated hydrogel, the initially defined porous architecture appeared less distinct, suggesting structural modification of the matrix during bacterial growth. One possible explanation is, as previously described, the enzymatic degradation of the gelatin component by extracellular proteases produced by *P. aeruginosa*, which has previously been associated with the degradation of protein-based matrices and may facilitate bacterial migration through the hydrogel [37]. As the gelatin component is degraded, the crosslinked alginate network may become the primary contributor to the remaining structural integrity of the matrix. Additionally, bacterial infiltration and associated matrix remodeling may alter the local pore architecture, potentially resulting in the loss of the well-defined porous morphology observed in the non-inoculated hydrogel.

A similar morphology was observed after 48 h of incubation (Figure 3C), with the distinct porous architecture observed in the non-inoculated hydrogel being no longer readily apparent. Importantly, however, bacteria were clearly visible at the apical surface of the hydrogel. Rather than being present exclusively as individual cells, bacteria appeared to form localized clusters or microcommunities, indicating the possible presence of biofilm [38]. The SEM images were acquired from the apical surface of the hydrogel, corresponding to the initial site of bacterial inoculation. The possible presence of bacterial clusters at the apical surface of the hydrogel structure therefore suggests that *P. aeruginosa* could be able to further migrate from the inoculation surface into the matrix and establish biofilm communities within the hydrogel. However, as the SEM images were acquired from the apical surface only, these observations do not provide clear evidence of bacterial infiltration into the hydrogel interior.

Overall, the SEM observations demonstrate that the hydrogel matrix supported bacterial growth and may support matrix infiltration and development of localized *P. aeruginosa* communities over 48 h. The progressive alteration of the hydrogel morphology during bacterial incubation further suggests that the matrix undergoes structural remodeling during biofilm development while retaining sufficient integrity to support bacterial colonization. These findings validate that the gelatin–alginate hydrogel supports the development of microcolonies and potentially biofilm formation and therefore appears as a suitable three-dimensional matrix for subsequent evaluation of antimicrobial treatments against both developing and established *P. aeruginosa* biofilms. Cross-sectional imaging would be required to directly evaluate the extent of bacterial infiltration within the three-dimensional hydrogel; however, the objective of the present SEM analysis was to confirm bacterial growth and microcolony formation on the hydrogel surface.

### 2.4. Antimicrobial Evaluation

To evaluate the suitability of the hydrogel model as an *in vitro* platform for assessing wound dressings and other topical antimicrobial treatments, different treatment strategies were investigated against both developing and established *P. aeruginosa* biofilms. The selected treatments were chosen to represent distinct antimicrobial approaches. TOB, an aminoglycoside antibiotic with well-established activity against *P. aeruginosa*, was included as a potent antibiotic treatment. Although aminoglycosides are effective against *P. aeruginosa*, their systemic use is limited by adverse effects, including nephrotoxicity, supporting the use of local administration for topical applications [39]. EPL was selected as a non-antibiotic antimicrobial due to its cationic, water-soluble nature and reported activity against both Gram-negative and Gram-positive bacteria [40]. Finally, Prontosan^®^ was included as an established clinical wound-cleansing formulation containing the antimicrobial agent polyhexanide and the surfactant betaine, which are reported to facilitate biofilm disruption and prevention of biofilm reformation [41]. Untreated hydrogels served as the growth control (GC).

**Figure 4:**
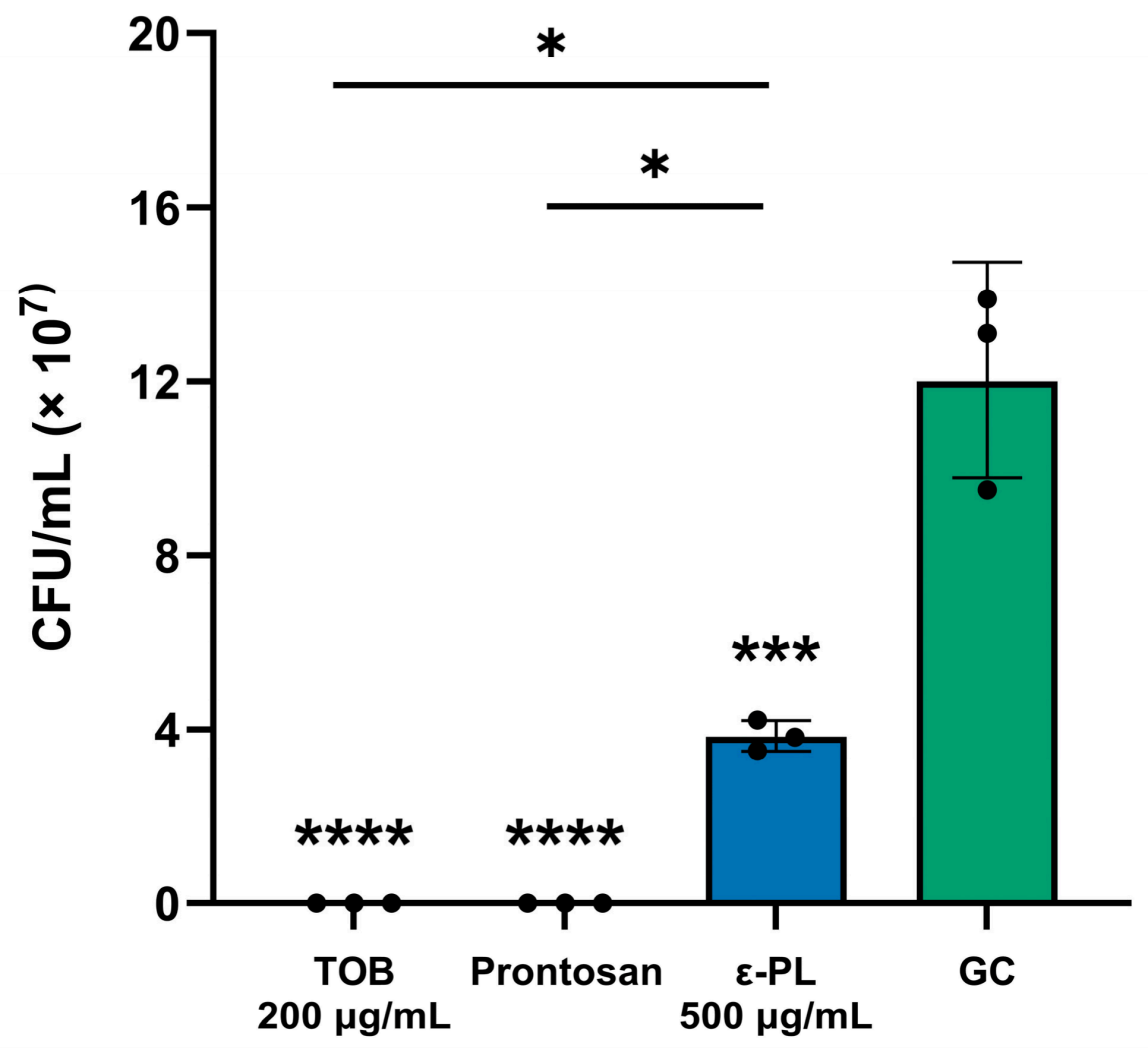
Evaluation of *P. aeruginosa* biofilm growth inhibition in the hydrogel wound model following 24 h treatment with 200 µg/mL tobramycin (TOB), Prontosan^®^, or 500 µg/mL ε-poly-L-lysine (EPL). Untreated hydrogels served as the growth control (GC). Bacterial growth was quantified by colony-forming unit (CFU) enumeration and expressed as CFU/mL (×10^7^). TOB: gray; Prontosan^®^: purple; EPL: blue; GC: green. Data are presented as means ± SDs; * = *p* < 0.05, *** = *p* < 0.001, and **** = *p* < 0.0001 (*n* = 3).

For evaluation of biofilm growth inhibition, the antimicrobial treatments were added following inoculation, and bacterial growth was quantified after 24 h. As shown in Figure 4, TOB at 200 µg/mL and Prontosan^®^ (B. Braun Melsungen AG, Melsungen, Germany) resulted in bacterial counts below the detection limit of the assay, with no colonies observed on the corresponding agar plates at the lowest dilution tested (10^−2^) (Figure S2). These results are therefore reported as no visible growth for the assay. EPL at 500 µg/mL resulted in 3.9 ± 0.4 × 10^7^ CFU/mL, compared with the growth control, which resulted in 12.2 ± 2.3 × 10^7^ CFU/mL. Although all treatments resulted in a significant reduction compared with the untreated growth control, TOB and Prontosan^®^ exhibited the greatest inhibitory effect. These results demonstrate that the hydrogel model was able to differentiate between treatments with distinct antimicrobial potencies.

**Figure 5:**
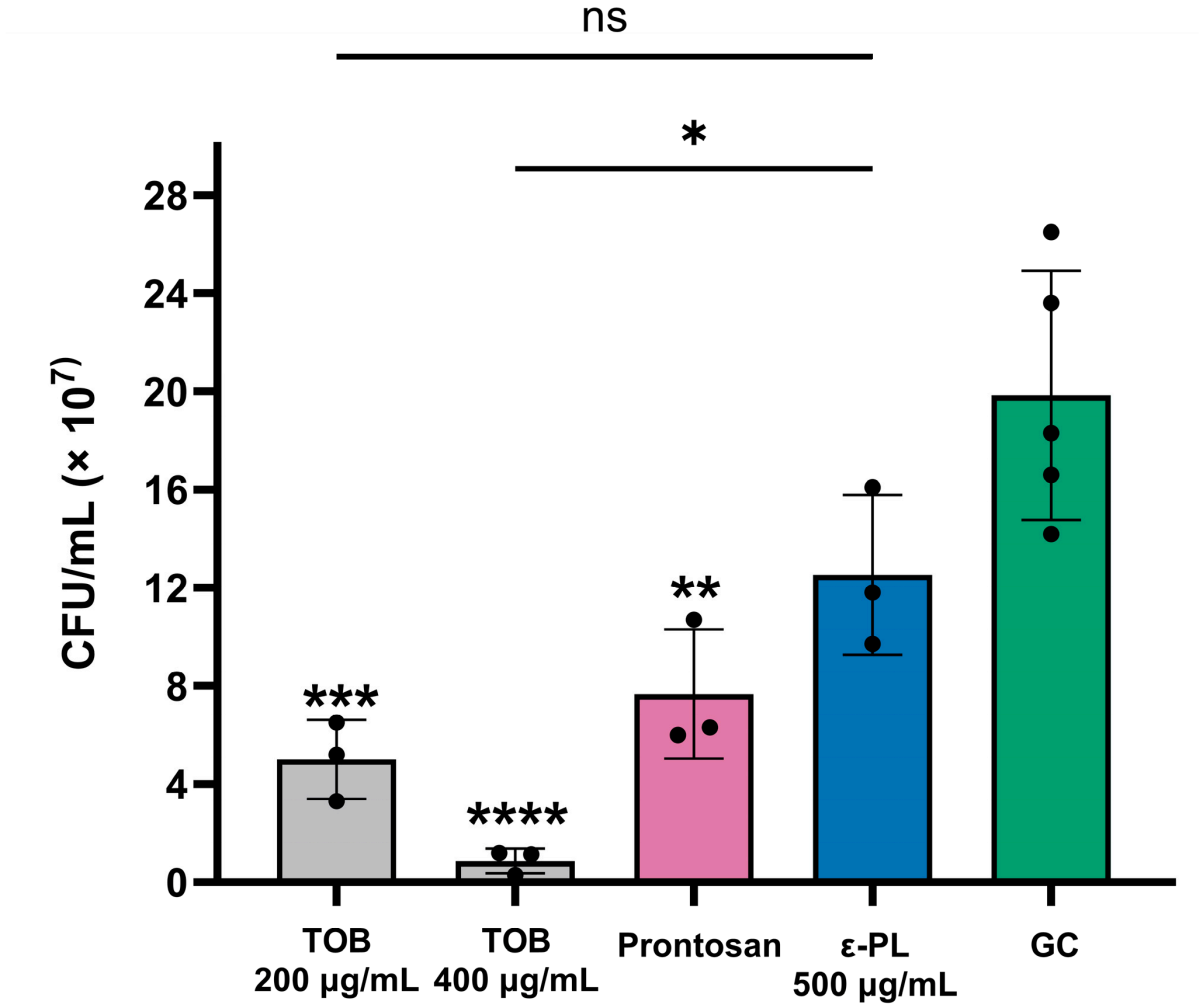
Evaluation of *P. aeruginosa* biofilm eradication in the hydrogel wound model. Biofilms were established for 24 h prior to treatment and subsequently incubated for an additional 24 h with 200 or 400 µg/mL tobramycin (TOB), Prontosan^®^, or 500 µg/mL ε-poly-L-lysine (EPL). Untreated hydrogels served as the growth control (GC). Bacterial growth was quantified by colony-forming unit (CFU) enumeration and expressed as CFU/mL (×10^7^). TOB: gray; Prontosan^®^: purple; EPL: blue; GC: green. Data are presented as means ± SDs; * = *p* < 0.05, ** = *p* < 0.01, *** = *p* < 0.001, and **** = *p* < 0.0001 (*n* = 3–5).

The ability of the model to evaluate eradication of established biofilms was subsequently investigated. Biofilms were allowed to develop for 24 h prior to addition of the antimicrobial treatments, followed by a further 24 h incubation period. Under these conditions, TOB again demonstrated the greatest reduction in recovered viable bacterial counts. TOB at 200 µg/mL resulted in a final bacterial growth of 5.0 ± 1.6 × 10^7^ CFU/mL, whereas increasing the concentration to 400 µg/mL further reduced the recovered bacterial count to 0.9 ± 0.5 × 10^7^ CFU/mL (Figure 5). The untreated control contained 19.8 ± 5.1 × 10^7^ CFU/mL after the corresponding 48 h incubation period. This was consistent with the limited bacterial growth observed visually in the corresponding wound models (Figure S3). Prontosan^®^ resulted in 7.6 ± 2.6 × 10^7^ CFU/mL, which still resulted in lower growth than EPL, which exhibited the lowest eradication efficacy, with a total growth of 12.5 ± 3.3 × 10^7^ CFU/mL. TOB at both 200 and 400 µg/mL was significantly more effective than the treatment with EPL. These lower final bacterial counts following TOB application at 200 and 400 µg/mL compared with the untreated control at 24 h suggest that the treatments reduced the viable bacterial population relative to the 24 h untreated reference. However, because the bacterial burden was not measured immediately before treatment in the same hydrogels, these results should not be interpreted as direct evidence of complete eradication of the established biofilm but act as a suggestion that the treatment achieves more than simply inhibiting further growth.

It should be noted that each treatment was administered only once in the present model. This is particularly relevant for Prontosan^®^, which is typically applied repeatedly during clinical wound management to facilitate removal and control of biofilms [41]. Consequently, the present experiments should be regarded as a proof-of-concept evaluation of the ability of the model to discriminate between antimicrobial treatments rather than as a direct comparison of clinical treatment efficacy. The selected treatments were deliberately chosen to encompass different antimicrobial strategies, ranging from a potent conventional antibiotic (TOB) to a non-antibiotic cationic polymer (EPL) and an established topical wound-care formulation (Prontosan^®^).

Conventional evaluation of antimicrobial wound dressings frequently relies on agar-based assays, including disc diffusion assays [42,43,44]. Such approaches primarily provide information on growth inhibition surrounding the treatment and consequently do not directly assess the ability of a formulation to eradicate an established biofilm. Furthermore, diffusion through agar can influence the apparent antimicrobial activity of wound dressings containing nanoparticles, polymers, or other larger components [45]. Limited diffusion may therefore result in an underestimation of the antimicrobial potential of formulations whose active components do not readily diffuse through the agar matrix.

Alternative approaches involve solubilization or extraction of wound dressing materials followed by evaluation against biofilms grown in static 96-well plate models. While these approaches can provide quantitative antimicrobial data, conventional microtiter models generally involve biofilms formed on rigid, planar surfaces and thus do not reproduce the three-dimensional and mechanically soft environment of a wound. In addition, biofilms in such models are typically established at a liquid–liquid interface, whereas wound biofilms can develop within or at the surface of hydrated, semi-solid tissue [8].

More complex three-dimensional models, such as the collagen-based layered wound model reported by Thaarup et al. (2023), provide a more physiologically relevant environment and can incorporate features such as oxygen and pH gradients [12]. However, the increased complexity of such systems also increases experimental time, material requirements, and methodological demands. For the initial screening of the biofilm eradication efficacy of wound dressing formulations, a simpler intermediate-complexity model may therefore provide a useful balance between physiological relevance and experimental practicality.

The present hydrogel model provides such an intermediate platform. From initiation of the experiment to final bacterial quantification, the model can be completed within approximately five days while providing a hydrated, three-dimensional and mechanically soft matrix into which *P. aeruginosa* can infiltrate and form biofilm communities. Nutrient availability is provided through static diffusion from the underlying medium, while the use of a wound-relevant matrix and medium provides a more representative environment than conventional two-dimensional microtiter models. Importantly, the model enables treatment of both developing and established biofilms and subsequent quantitative determination of bacterial survival by CFU enumeration. Moreover, the present model represents a cost-effective alternative to previously reported collagen-based wound models (Table S1). A comparison of estimated material costs per insert/well with both the layered collagen wound model by Thaarup et al. (2023) (Table S2) and the collagen model by Werthèn et al. (2009) (Table S3) showed that the material costs of the current system were lower [10,12]. Specifically, the estimated material costs were €1.82 per model for the present system, compared with €4.10 for the model described by Thaarup et al. and €2.20 for the model described by Werthèn et al. This difference was primarily associated with the use of gelatin rather than collagen as the main matrix component. It should be noted that the transwell insert itself constituted the largest individual cost component in both the present model and the model described by Thaarup et al. In addition to the lower material costs, the model relies on standard laboratory equipment, readily accessible materials, and simple preparation procedures, which may facilitate implementation in a broader range of laboratory settings.

The model nevertheless has several limitations. It currently represents a monospecies biofilm and does not reproduce the polymicrobial communities commonly associated with chronic wounds [8,12,46]. Moreover, the present model was established using the laboratory strain *P. aeruginosa* PAO1, which was selected because it is a well-established and reproducible biofilm-forming strain. However, the use of a single laboratory strain represents a limitation with respect to the broader applicability of the model to clinically relevant wound infections. Future studies should therefore also include additional clinically relevant wound isolates, including strains obtained from chronic wound infections, to further validate the applicability of the proposed platform. Furthermore, although the model provides a three-dimensional matrix environment, oxygen and pH gradients within the hydrogel were not characterized. These factors may substantially influence biofilm physiology and antimicrobial susceptibility and should therefore be considered in future development of the model. Nevertheless, incorporating these features was beyond the scope of the present study. The current results demonstrate that the hydrogel system can support *P. aeruginosa* growth and microcolony formation and can distinguish differences in the efficacy of antimicrobial treatments against both developing and established biofilms, supporting its potential as a practical screening platform for topical antimicrobial and wound-dressing formulations.

## 3. Conclusions

In this study, a three-dimensional gelatin–alginate hydrogel wound model was developed and characterized as an *in vitro* platform for investigating *P. aeruginosa* biofilm growth and antimicrobial efficacy. The hydrogel matrix supported bacterial growth and microcolony formation within the matrix while maintaining sufficient structural integrity for quantitative bacterial recovery. The model enabled differentiation between antimicrobial inhibition and eradication of established biofilms. Overall, the developed model provides a simple and practical intermediate-complexity platform for evaluating topical antimicrobial and wound-dressing formulations against *P. aeruginosa* biofilm, while offering greater structural relevance than the conventional two-dimensional assays.

## 4. Materials and Methods

### 4.1. Materials

Sodium alginate, gelatin type A (~300 Bloom, porcine skin), chloroform (≥99.8%), phosphate-buffered peptone water (GranuCult^®^, Sigma-Aldrich St. Louis, MO, USA), calcium chloride, trisodium citrate dihydrate, tobramycin, glutaraldehyde (25%, *v*/*v*; electron microscopy-grade), Luria–Bertani (Lennox) broth, and absolute ethanol were purchased from Sigma-Aldrich (St. Louis, MO, USA). Dulbecco’s phosphate-buffered saline (DPBS; calcium- and magnesium-free) and fetal bovine serum (FBS) were purchased from Thermo Fisher Scientific (Waltham, MA, USA). Microbial transglutaminase (mTG; 1000 U/g) was purchased from MarkNature (Fullerton, CA, USA). Prontosan^®^ wound irrigation solution was purchased from B. Braun Melsungen AG (Melsungen, Germany). ε-Poly-L-lysine (EPL) was purchased from Handary S.A. (Brussels, Belgium). All chemicals and reagents were used as received.

### 4.2. Bacterial Culture Conditions

*P. aeruginosa* laboratory strain PAO1 wildtype (wt) was used throughout this study. For preparation of overnight cultures, PAO1 wt was streaked from a cryogenic stock stored at −80 °C onto Luria–Bertani (Lennox) (LB) agar and incubated for 24 h in 37 °C. A single colony-forming unit (CFU) was subsequently inoculated into 10 mL LB medium and incubated for 16–18 h at 37 °C with shaking at 150 rpm to reach the stationary phase.

### 4.3. Hydrogel Preparation

The day before casting the hydrogels in transwells, sodium alginate was dissolved in sterile Milli-Q at a final concentration of 4% (*w*/*v*) with initial vigorous stirring (1000 RPM). When all sodium alginate was added, the stirring was adjusted to 60 RPM, and the solution was stirred overnight (O/N). The next day, SWF consisting of 1:1 autoclaved 0.1% (*w*/*v*) phosphate-buffered peptone water in saline and fetal bovine serum (FBS) was heated to 37 °C and stirred at 180 RPM, and GA was added to a final concentration of 10% (*w*/*v*). The solution was then stirred at 37 °C for 1 h. When both polymer solutions were homogeneous, the GA and sodium alginate were mixed at 1:1 at 37 °C for a few minutes to reach a final concentration of 5 and 2% (*w*/*v*) of GA and sodium alginate, respectively. Chloroform was added to a final concentration of 0.5% (*v*/*v*) to sterilize the solution [47]. The hydrogel solution was added to 24-well transwell inserts (Greiner bio-one ThinCert^®^, Kremsmünster, Austria, 1 µm pore size) in a volume of 100 µL while avoiding formation of bubbles during aspiration. Following transfer, the chloroform was allowed to evaporate according to the procedure described by Dogan and Dufva (2022) before thermal crosslinking was performed. This evaporation step was included to remove chloroform prior to subsequent further crosslinking and bacterial inoculation [47].

Initially, the hydrogels in transwells were incubated for 30 min at 4 °C to enable the thermal crosslinking of GA. Afterwards, 1000 µL and 100 µL of mTG (10% (*w*/*v*) in DPBS, 0.45 µm filtered) were added basolaterally and apically, respectively, and then incubated at room temperature for 1 h to irreversibly crosslink GA. This was followed by a three-time wash with DPBS basolaterally and apically. Next, CaCl_2_ (1.5% (*w*/*v*) in Milli-Q, 0.45 µm filtered) was likewise added basolaterally and apically for 10 min followed by important washing steps consisting of 3 washes with sterile Milli-Q and DPBS. The crosslinked hydrogels were left in DPBS at 4 °C until usage.

### 4.4. Stability Testing of the Hydrogels

For stability testing, the hydrogels were prepared as indicated in Section 4.3. Hydrogel Preparation. Hydrogels were removed from the transwells using 27G needles for evaluation of the following.

#### 4.4.1. Loss on Drying

For determination of loss on drying (LOD), hydrogels were placed in Petri dishes and incubated at 37 °C for 48 h. The weight and diameter of the hydrogels were recorded at 0, 0.5, 1, 2, 3, 24, and 48 h. LOD was calculated according to the following equation:

(1) $$LOD\;\left(\%\right)=\frac{W_{0}-W_{t}}{W_{0}}\times 100\%\;$$

where *W_t_* is the weight of the drying sample at a predetermined time and *W_0_* is the initial wet weight at time 0 h.

#### 4.4.2. Swelling Ratio

For determination of the swelling ratio (SR), hydrogels were first dried at 37 °C for 24 h and subsequently immersed in PBS at 37 °C. At 0, 0.5, 1, 2, 3, 24, and 48 h, the hydrogels were removed from the PBS, and excess surface liquid was gently removed before weighing. The SR was calculated according to the following equation:

(2) $$SR\;\left(\%\right)=\frac{W_{t}-W_{0}}{W_{0}}\times 100\%\;$$

where *W_t_* is the weight of the swollen sample at a predetermined time and *W_0_* is the initial dry weight at time 0 h.

#### 4.4.3. Compressive Mechanical Properties

To characterize the mechanical properties of the hydrogel matrix, hydrogels were prepared as described in Section 4.3. Hydrogel Preparation and incubated overnight at 37 °C with SWF in the basolateral compartment. Prior to mechanical testing, the hydrogels were carefully removed from the transwell inserts using a 27 G syringe. The hydrogel samples had initial height and diameters of 2.6 ± 0.4 mm and 6.1 ± 0.3 mm (*n* = 6), respectively.

Compression testing was performed using a texture analyzer (XT Plus, Stable Micro Systems Ltd., Godalming, Surrey, UK). The pre-test and test speeds were set to 1.0 and 0.5 mm/s, respectively, with a trigger force of 5 g. Mechanical behavior was initially evaluated by compressing the hydrogels to 50% of their initial heights for two consecutive compression cycles. In a separate measurement, hydrogels were compressed until mechanical failure. Each measurement was performed in triplicate. The compressive stress (σ), was calculated from the applied force according to

(3) $$\sigma =\frac{F}{A}$$

where *F* is the applied force (N) and *A* is the initial cross-sectional area of the hydrogel (m^2^), resulting in stress expressed in Pa. The compressive strain (ε) was calculated as:

(4) $$\varepsilon =\frac{∆h}{h_{0}}$$

where ∆h is the change in sample height and $h_{0}$ is the initial sample height. Strain was expressed as percentage compression by multiplying ε by 100%.

The compressive modulus (*E_c_*) was determined from the slope of the linear region of the stress–strain curve:

(5) $$E_{c}=\frac{∆\sigma }{∆\varepsilon }$$

### 4.5. Biofilm Formation

Biofilm formation in the hydrogel in the transwells was initiated by adjusting *P. aeruginosa* PAO1 wt in the mid-log phase to an OD_600_ of 0.0001 in sterile saline. The transwells were removed from the well plates and transferred to a petri dish, where 100 µL of the inoculum was added, and the transwells were incubated for 30 min at 37 °C without shaking. Next, the inoculum was removed, and the transwells were added to a new 24-well plate containing pre-warmed SWF in the basolateral compartment. The transwell system was incubated at 37 °C at 50 RPM for a period of 24–48 h depending on evaluation of either inhibition or eradication of growth.

### 4.6. Inhibition of Biofilm Growth

For evaluation of biofilm growth inhibition, the inoculum was removed after an initial 30 min of incubation, and 50 µL of the antimicrobial sample (200 µg/mL TOB, Prontosan^®^, or 500 µg/mL EPL) was subsequently added. The system was then incubated for an additional 24 h at 37 °C and 50 RPM, after which biofilm formation was quantified. A growth control was included with no treatment added.

### 4.7. Eradication of Biofilm Growth

For evaluation of biofilm eradication, initially, the inoculum was aspirated after 30 min, and the transwells were further incubated for 24 h at 37 °C and 50 RPM. Following biofilm formation, the transwells were washed basolaterally with sterile saline to remove non-adherent bacteria, after which 50 µL of the antimicrobial treatment was added (200 µg/mL TOB, 400 µg/mL TOB, Prontosan^®^, or 500 µg/mL EPL). The antimicrobial concentrations were selected for proof-of-concept evaluation of the model, with TOB being tested at 200 and 400 µg/mL to assess whether the model could differentiate between two concentrations of the same antibiotic, and EPL being tested at 500 µg/mL based on preliminary experiments and its previously determined antimicrobial activity against *P. aeruginosa* PAO1. The system was subsequently incubated with new SWF added basolaterally for an additional 24 h at 37 °C and 50 RPM, and the remaining biofilm was quantified. Likewise, a GC was included for eradication of growth.

### 4.8. Biofilm Quantification by Colony-Forming Unit Enumeration

For quantification of bacterial growth, the transwells were first washed basolaterally with saline. The hydrogels were then removed from the transwells by carefully cutting along the edges using a 27G syringe needle and transferred directly into Eppendorf tubes. Subsequently, 1 mL of autoclaved 4% (*w*/*v*) trisodium citrate dihydrate prepared in Milli-Q water was added to each tube. For samples where the gelatin matrix was not fully dissolved, mechanical mixing using a pipette tip was performed to increase the exposed surface area and disrupt the remaining matrix as thoroughly as possible. The samples were shaken at 150 RPM for 50 min to dissolve the alginate matrix. The resulting suspension was centrifuged at 10,000 ×*g* for 5 min to remove any residual treatment and the trisodium citrate dihydrate, after which the supernatant was removed and the bacterial pellet was resuspended in 1 mL of DPBS.

The resulting bacterial suspension was serially diluted up to 10^−6^, depending on the treatment group. For each sample, three appropriate dilutions were plated on LB agar and incubated overnight at 37 °C. The number of CFU was subsequently enumerated and calculated from dilution and expressed as total CFU/mL.

### 4.9. Method Validation and Hydrogel Optimization

To verify that trisodium citrate dihydrate did not adversely affect bacterial viability during hydrogel dissolution, a citrate toxicity control was performed. A 100 µL bacterial suspension adjusted to an OD_600_ of 0.001 was added to 5 mL of the 4% (*w*/*v*) trisodium citrate dihydrate solution and incubated for 50 min with shaking. In parallel, an equivalent bacterial suspension was incubated in DPBS under the same conditions. Following incubation, both suspensions were serially diluted and plated on LB agar. Plates were incubated at 37 °C for 24 h, after which the number of CFU was enumerated and compared between the two conditions.

Following evaluation of the potential effect of trisodium citrate dehydrate on bacterial viability, the bacterial recovery efficiency of the hydrogel recovery procedure was determined. Hydrogels were prepared according to Section 4.3. Hydrogel Preparation. A 100 µL inoculum of *P. aerugonosa* PAO1 suspension with an OD_600_ of 0.1, corresponding to approximately 2 × 10^7^ CFU/mL, was applied to each hydrogel-containing transwell insert. Immediately following inoculation, the samples were processed according to the bacterial recovery procedure described in Section 4.8. Biofilm Quantification by Colony-Forming Unit Enumeration. The recovered bacterial suspensions were serially diluted and plated on LB agar at dilutions of 10^−3^, 10^−4^, and 10^−5^. As reference, 100 µL of the same inoculum was directly serially diluted and plated on LB agar using the same plating procedure. All plates were incubated overnight at 37 °C, after which colonies were enumerated. Four independent replicates were evaluated for both the hydrogel recovery and control groups.

Recovery efficiency (RE) was calculated relative to the direct-plating control according to Equation (6):

(6) $$RE=\frac{CFU_{recovered}}{CFU_{control}}\times 100\%$$

where *CFU_recovered_* represents the mean CFU concentration recovered following processing of the hydrogel samples and *CFU_control_* represents the mean CFU concentration obtained from the corresponding direct-plating control.

During optimization of the hydrogel matrix for biofilm formation, different concentrations of CaCl_2_ were evaluated as crosslinking agent for sodium alginate. Final CaCl_2_ concentrations of 1.0, 1.5, 2.0, and 2.5% (*w*/*v*) were investigated while maintaining the remaining hydrogel preparation and biofilm cultivation conditions as described in Section 4.3. Hydrogel Preparation and Section 4.5. Biofilm Formation. Following 24 h of biofilm growth, the bacterial cells were harvested and quantified according to the procedure described in Section 4.8. Biofilm Quantification by Colony-Forming Unit Enumeration.

### 4.10. Hydrogel Sample Preparation and Imaging Using Scanning Electron Microscopy

For SEM analysis, hydrogel samples representing 0 h, 24 h, and 48 h of bacterial growth were prepared. Hydrogels were prepared in transwells and inoculated as described in Section 4.3. Hydrogel Preparation and 4.5. Biofilm Formation. Samples at 0 h were not inoculated with bacteria, whereas the 24 h and 48 h samples were inoculated with *P. aeruginosa*.

At the respective time points of 0, 24 and 48 h, the hydrogels were fixed by incubation in 2.5% (*v*/*v*) glutaraldehyde in DPBS (EM grade) for 24 h at room temperature. Following fixation, the samples were washed with DPBS and dehydrated through a graded ethanol series of 30, 50, 70, 80, 90, 96 and 100% (*v*/*v*), followed by two additional 100% ethanol steps. Each dehydration step was performed for 15 min. The samples were subsequently removed from the transwells and stored in 100% ethanol until critical point drying (CPD).

To minimize structural deformation during drying, the samples were dried using CPD (Leica EM CPD300, Leica Microsystems GmbH, Wetzlar, Germany). The dried samples were sputter-coated with a 10 nm platinum layer at 20 mA (Leica EM ACE600 Sputter coater, Germany). The coated samples were examined using SEM imaging (Quanta FEG 200, FEI Company, Hillsboro, OR, USA) at an accelerating voltage of 5 kV with a spot size of 3. SEM imaging was performed to evaluate the distribution and infiltration of bacterial cells within the hydrogel matrix.

### 4.11. Data and Statistical Analysis

All data are expressed as means ± SDs, unless otherwise stated. For comparison of more than two mean values, a one-way ANOVA with Tukey’s multiple comparison was used; otherwise unpaired Student’s *t*-test was employed, where *p*-values were considered statistically significant when below 0.05. Graphs and statistical tests were performed using GraphPad Prism version 11.0.1 (GraphPad Software, San Diego, CA, USA).

## Data Availability

The original contributions presented in this study are included in the article/Supplementary Materials. Further inquiries can be directed to the corresponding authors.

## Supplementary Materials

The following supporting information can be downloaded at: https://www.mdpi.com/article/10.3390/gels12100934/s1. Figure S1. Uniaxial compression testing of three individual hydrogel samples subjected to two consecutive compression cycles of 50% compressive strain; Figure S2. Compression of three individual hydrogel samples until mechanical failure; Figure S3. Optimization of the hydrogel wound model using increasing concentrations of CaCl_2_ as the alginate crosslinking agent; Figure S4. Representative images of bacterial growth in the hydrogel wound model following treatment for biofilm growth inhibition; Figure S5. Representative images of bacterial growth in the hydrogel wound model following treatment of established biofilms; Table S1. Estimated cost of established wound model estimated per transwell insert; Table S2. Estimated cost of the “Collagen-based layered chronic wound model” from Thaarup I. C. et al. (2023) [12]. The estimation is based on provided concentrations, and volumes are calculated from Chen X. el al. (2021) [48]. Representative materials are found from suppliers and described as estimated cost for material. Not all materials were available for purchase; Table S3. Estimated cost of the “in vitro model of bacterial infections in wounds and other soft tissues” by Werthén M. et al. (2009) [10]. The estimation is based on provided concentrations and volumes as well as representative materials found from suppliers.
