# Supplementary material for: A Cost-Effective Hydrogel-Based Wound Model for Quantitative Evaluation of Topical Treatments in Infected Wounds
Source: Gels. 2026 Oct 16;12(10):934. doi: 10.3390/gels12100934 (PMC13704363; doi:10.3390/gels12100934)
Supplement: Supplementary file 1 [file gels-12-00934-s001.zip › gels-4566285-supplementary.pdf]

A Cost-Effective Hydrogel-Based Wound Model for Quantitative Evaluation of Topical Treatments in Infected Wounds

Amalie Kjær Andresen[1]*, Emilia Gaertner [1], Marie Karen Tracy Hong Lin[2], Claus Moser[3,4], Lars Jelsbak[5] and Line Hagner Nielsen[1]*

[1] Department of Health Technology, Technical University of Denmark, Ørsteds Plads 345C, 2800 Kgs. Lyngby, Denmark; akjan@dtu.dk, gaertner.emilia@gmail.com, lihan@dtu.dk

[2] National Centre for Nano Fabrication and Characterization, Technical University of Denmark, Fysikvej 307, 2800 Kgs. Lyngby, Denmark; mktracy@dtu.dk

[3] Department of Clinical Microbiology, Rigshospitalet, Copenhagen University Hospital, Blegdamsvej 9, 2100, Copenhagen Ø, Denmark; moser@dadlnet.dk

[4] Department of Immunology and Microbiology, Costerton Biofilm Center, University of Copenhagen, Blegdamsvej 3B, Copenhagen, Denmark; moser@dadlnet.dk

[5] Department of Biotechnology and Biomedicine, Section for Microbial and chemical ecology, Technical University of Denmark, Søltofts Plads, 221, 260, 2800 Kgs. Lyngby, Denmark; lj@bio.dtu.dk

* Correspondence: akjan@dtu.dk, lihan@dtu.dk

Supporting information

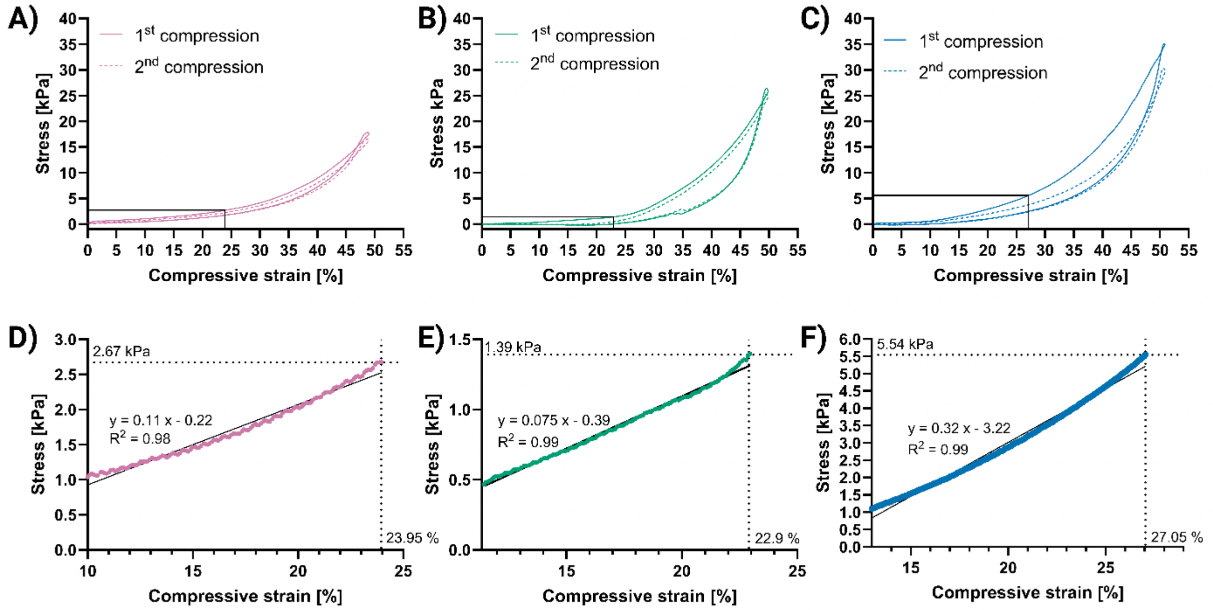

Figure S1. Uniaxial compression testing of three individual hydrogel samples subjected to two consecutive compression cycles of 50% compressive strain. Colors correspond to the individual replicates shown in **Figure 3D.** In A-C, solid lines represent the first compression cycle, whereas dashed lines represent the second compression cycle. The initial linear regions of the stress-strain curves in A-C were analyzed by linear regression, as shown in D-F, and the resulting slopes were used to determined the compressive modulus.

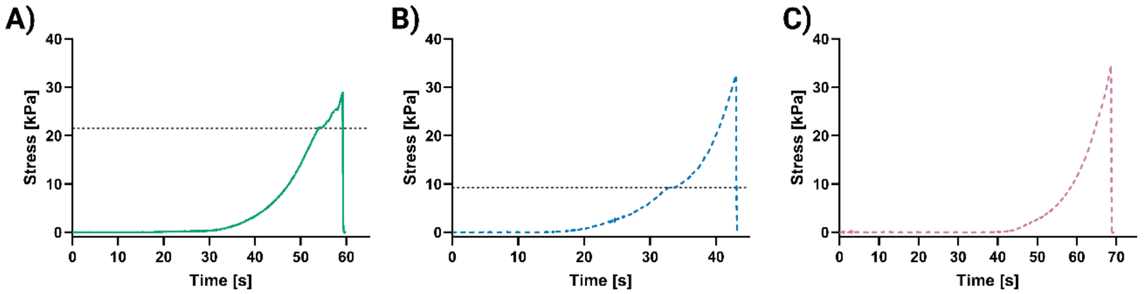

Figure S2. Compression of three individual hydrogel samples until mechanical failure. Failure points indentified on A) and B) with no apparent failure point found in C). Colors correspond to the individual replicates shown in **Figure 3E.**

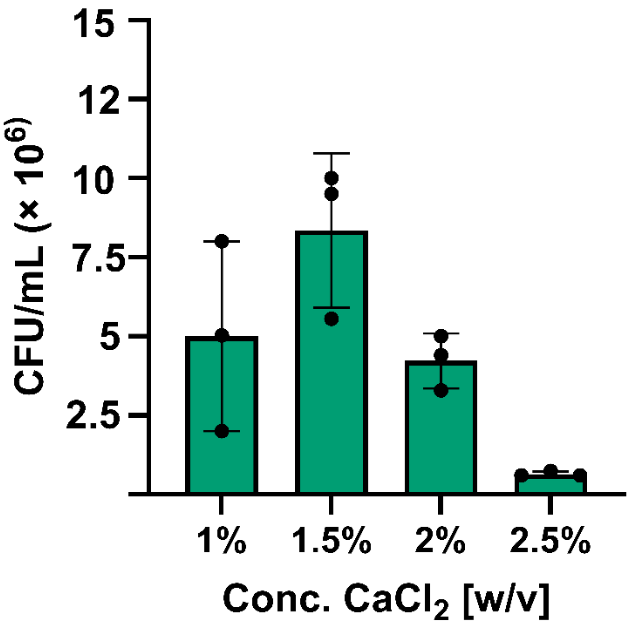

Figure S3. Optimization of the hydrogel wound model using increasing concentrations of $CaCl_2$ as the alginate crosslinking agent. Bacterial recovery was evaluated by CFU enumeration following 24 h incubation with *P. aeruginosa*. $CaCl_2$ concentrations of 1.0, 1.5, 2.0, and 2.5% (w/v) were investigated.

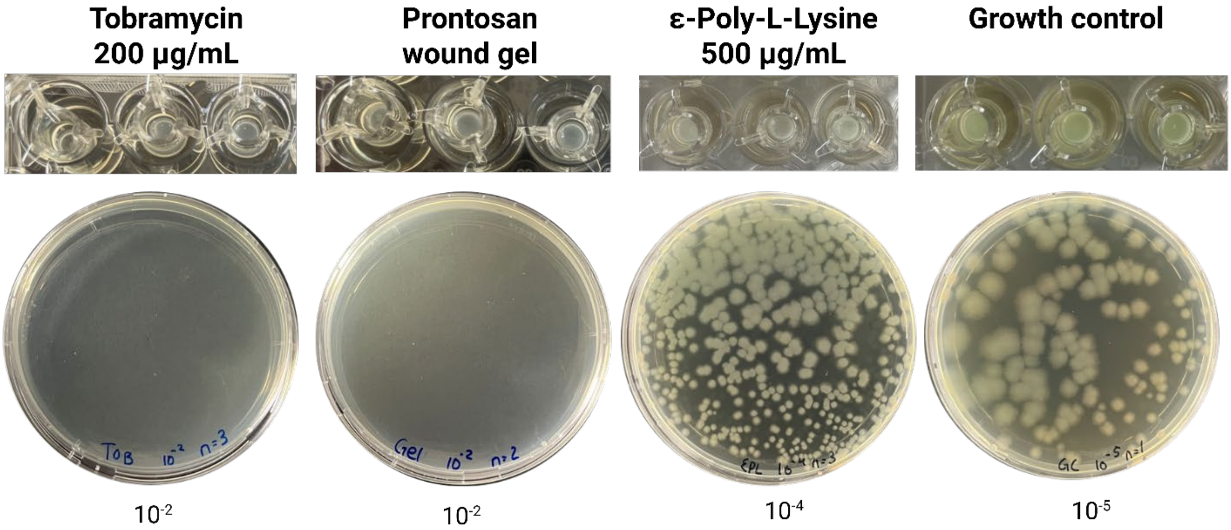

Figure S4. Representative images of bacterial growth in the hydrogel wound model following treatment for biofilm growth inhibition. Hydrogels were treated with 200 µg/mL tobramycin (TOB), Prontosan®, or 500 µg/mL ε-poly-L-lysine (EPL), with untreated hydrogels serving as the growth control. Representative agar plates from relevant serial dilutions are shown, corresponding to the lowest dilution with countable colonies.

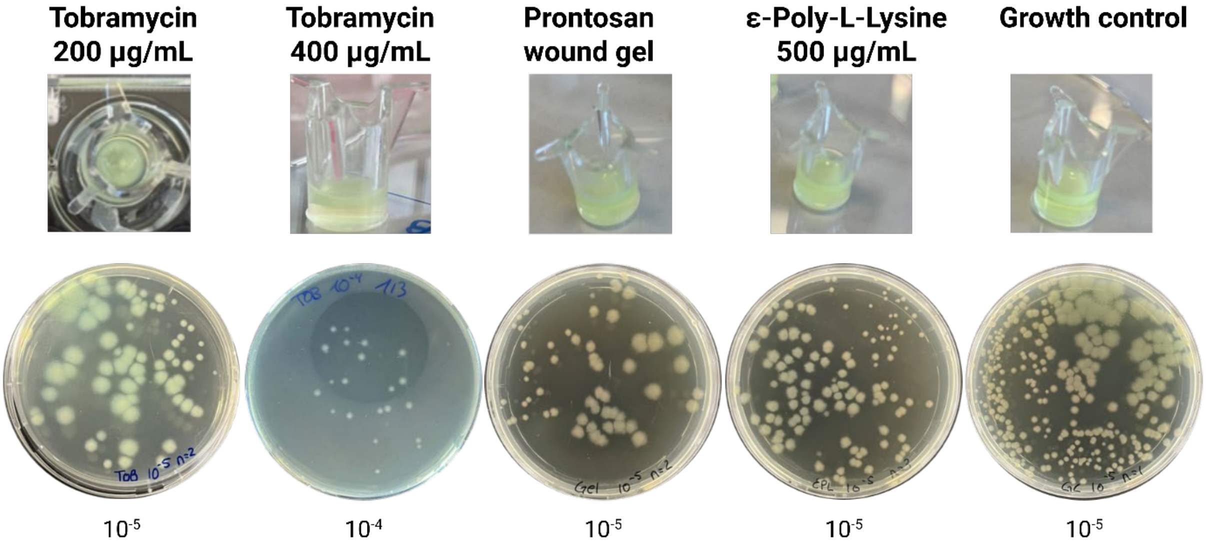

Figure S5. Representative images of bacterial growth in the hydrogel wound model following treatment of established biofilms. Biofilms were treated with 200 or 400 µg/mL tobramycin (TOB), Prontosan®, or 500 µg/mL ε-poly-L-lysine (EPL), with untreated hydrogels serving as the growth control. Representative agar plates from relevant serial dilutions are shown, corresponding to the lowest dilution with countable colonies.

Table S1. Estimated cost of established wound model estimated per transwell insert. Cas numbers are indicated if available from supplier. The estimated cost is calculated from concentrations and volumes used in the model.

**Material**	**Supplier**	**Cas no.**	**Cost per Material (weight)**	**Used per 20 models**	**Estimated cost per transwell**
Sodium alginate	Sigma-Aldrich	9005-38-3	€131.16 / 1 kg	100 mg	€0.0007
Gelatine type A	Sigma-Aldrich	9000-70-8	€54.95 /100 g	200 mg	€0.0028
Phosphate-buffered peptone water	Sigma-Aldrich	-	€85.43 / 500 g	15 mg	€0.00013
Calcium chloride	Sigma-Aldrich	10043-52-4	€78.75/ 500 g	0.33 mg	€0.0013
Trisodium citrate dihydrate	Sigma-Aldrich	6132-04-3	€331.58 / 1 kg	800 mg	€0.0133
Fetal bovine serum	Thermo Fisher Scientific	-	€893.12 /500 mL	20 mL	€1.791
Microbial transglutaminsase	MarkNature	80146-85-6	€158.84 / 1 kg	2 g	€0.0159
**Total**					**€1.82**
24-well transwell inserts	Greiner bio-one		€318.47 /48 units	1 unit	€6.63
Total including transwell					€8.45

Table S2. Estimated cost of the "Collagen-based layered chronic wound model" from Thaarup I. C. et al. (2023) [1]. The estimation is based on provided concentrations, and volumes are calculated from Chen X. el al. (2021) [2]. Representative materials are found from suppliers and described as estimated cost for material. Not all materials were available for purchase.

Material	Example supplier/catalog no.	Estimated cost for material	Estimated amount per transwell	Estimated cost per transwell
Peptone water	Oxoid™ Peptone Bacteriological OXLP0037B	€579.98/kg	7.7 mg	€0.005
Cattle serum	SSI Diagnostica	€1,000/L	112 µL	€0.1
Laked horse blood	Thermo Fisher Scientific R54072	€1,217.8/L	8.3 µL	€0.01
Alpha-amylase	Sigma-Aldrich A6255	€6,676.6/g	140 U/L	€0.0004
Rat tail tendon collagen (type 1)	Corning Inc.	€2.53/mg	0.77 mg	€1.9
Peptone water	Oxoid™ Peptone Bacteriological OXLP0037B	€579.98/kg	7.7 mg	€0.005
Pig fat	-	-	26.5 mg	-
Fetal bovine serum	Thermo Fisher Scientific	€893.12 (500 mL)	20 mL	€1.8
phosphate-buffered peptone water	Sigma-Aldrich	€85.43 (500g)	15 mg	€0.00013
**Total**				**€ 4.1**
12-well transwell inserts 0.4 um pore size	Fischer Scientific	€450/48 units	1 unit	€9.4
Total including transwell				€13.5

Table S3. Estimated cost of the "*in vitro* model of bacterial infections in wounds and other soft tissues" by Werthén M. et al. (2009) [3]. The estimation is based on provided concentrations and volumes as well as representative materials found from suppliers.

**Material**	**Example supplier/catalog no.**	**Estimated cost for material (weight)**	**Estimated amount per well**	**Estimated cost per transwell**
Fetal bovine serum	Thermo Fisher Scientific	€893.12 (500 mL)	1 mL	€1.79
phosphate-buffered peptone water	Sigma-Aldrich	€85.43 (500g)	15 mg	€0.00013
Polymerized rat-tail collagen type I	Corning Inc.	€2.53/mg	0.4 mg	€1.01
**Total**				**€2.2**

References

1. Thaarup, I.C.; Lichtenberg, M.; Nørgaard, K.T.H.; Xu, Y.; Lorenzen, J.; Thomsen, T.R.; Bjarnsholt, T. A Collagen-Based Layered Chronic Wound Biofilm Model for Testing Antimicrobial Wound Products. *Wound Repair and Regeneration* **2023**, *31*, 500–515, doi:10.1111/WRR.13087.

2. Chen, X.; Lorenzen, J.; Xu, Y.; Jonikaite, M.; Thaarup, I.C.; Bjarnsholt, T.; Kirketerp-Møller, K.; Thomsen, T.R. A Novel Chronic Wound Biofilm Model Sustaining Coexistence of Pseudomonas Aeruginosa and Staphylococcus Aureus Suitable for Testing of Antibiofilm Effect of Antimicrobial Solutions and Wound Dressings. *Wound Repair and Regeneration* **2021**, *29*, 820, doi:10.1111/WRR.12944.

3. Werthén, M.; Henriksson, L.; Jensen, P.Ø.; Sternberg, C.; Givskov, M.; Bjarnsholt, T. An in Vitro Model of Bacterial Infections in Wounds and Other Soft Tissues. *APMIS* **2010**, *118*, 156–164, doi:10.1111/J.1600-0463.2009.02580.X.
